# Host interaction functions of LIC_10902 and LIC_20185 highlight the multifactorial nature of *Leptospira interrogans* pathogenesis

**DOI:** 10.3389/fcimb.2026.1988027

**Published:** 2026-10-16

**Authors:** João P. Gaspar, Julia E. L. Paixao, Matthew C. Surdel, Jenifer Coburn, Aline F. Teixeira, Ana L. T. O. Nascimento

**Affiliations:** 1 Laboratório de Desenvolvimento de Vacinas, Instituto Butantan, São Paulo, SP, Brazil; 2 Programa de Pós-Graduação Interunidades em Biotecnologia, Instituto de Ciências Biomédicas, Universidade de São Paulo, São Paulo, SP, Brazil; 3 Department of Medicine, Division of Infectious Diseases, Medical College of Wisconsin, Milwaukee, WI, United States

**Keywords:** FecR proteins, host-pathogen interaction, *Leptospira*, leptospirosis, pathogenesis

## Abstract

Leptospirosis is a neglected global zoonosis caused by pathogenic Leptospira, which rely on a vast repertoire of outer membrane proteins (OMPs) to mediate tissue colonization and immune evasion. Despite being usually associated with regulation of gene expression of iron transport system proteins, FecR domain-containing proteins seem to possess a multifunctional role for *Leptospira*. This study characterizes two FecR proteins from *L. interrogans* serovar Copenhageni, LIC_10902 and LIC_20185, previously predicted as hypothetical proteins. Target proteins were analysed in silico for cloning parameters obtention and structural analysis. Target protein genes were amplified from *L. interrogans* sv. Copenhageni genome, cloned in the pAE vector and recombinant proteins were produced in *Escherichia coli* BL21 Star (DE3) pLysS. Recombinant protein purification was achieved by metal affinity chromatography and purified recombinant proteins were structurally characterized by circular dichroism (CD), and their immunogenicity and role in host-pathogen interactions were evaluated by ELISA. Analysis shows these proteins are conserved among pathogenic species and suggested that both contain a β-sheet-rich FecR domain. Purified recombinant proteins were produced in *Escherichia coli*. *In vitro*, rLIC_20185 bound elastin and E-cadherin, while both proteins interacted with plasminogen (PLG), vitronectin (VT), and complement components C4b, C7, C8, and C9. Experiments with LIC_10902 fragments indicated that the FecR domain mediates C8, C9, and PLG binding. Furthermore, both proteins inhibited *in vitro* C9 polymerization, and their interaction with PLG enabled its activation into plasmin (PLA). Although production in non-pathogenic *L. biflexa serovar* Patoc (*Lb*P) did not increase resistance to normal human serum (NHS), our results collectively suggest that both proteins could mediate pathogen-host interactions and potentially contribute to tissue adhesion and immune evasion.

## Introduction

Leptospirosis is a neglected global zoonosis with an immense impact on public health, affecting human and animal populations in various regions of the world, especially in tropical and developing countries. It is estimated that the disease is responsible for more than 1 million infections annually, resulting in approximately 60,000 deaths worldwide (Costa et al., 2015). Caused by pathogenic spirochetes of the genus *Leptospira*, human infection usually occurs incidentally through direct or indirect contact of abrasions on the skin or mucous membranes with water and soil contaminated by the urine of reservoir animals (Haake and Levett, 2015).

Maintaining the complex epidemiological cycle of *Leptospira* requires a remarkable adaptive capacity from this pathogen. Pathogenic spirochetes, such as *L. interrogans*, must be able to survive for long periods under abiotic environmental pressures (Davignon et al., 2023) until an opportunity to invade and colonize the complex network of tissues of a mammalian host appear (Picardeau, 2017). To ensure the establishment of infection, *Leptospira* relies on remodeling of its gene expression and cell surface, preparing an array of proteins capable of interacting with the host’s defenses and tissues (Rajapakse et al., 2025).

The colonization of host tissues is a complex process, largely mediated by OMPs, which act not only by anchoring bacteria to the extracellular matrix (ECM) but also by interacting directly with epithelial and endothelial cell lineages (Takahashi et al., 2022). Based on their OMPs, pathogenic leptospires interact with various components of the host’s ECM, such as glycosaminoglycans (GAGs), cellular fibronectin (FC), laminin, and collagen fibers. In addition to ensuring adhesion, the extensive repertoire of surface proteins plays a dual role in virulence by interfering with the host’s plasma proteins, such as the components of the coagulation, fibrinolytic and complement systems, ensuring bacterial dissemination and evasion of the immune system (Daroz et al., 2021).

Although the role of proteins containing the FecR domain in *L. interrogans* is still poorly explored, work carried out by our research group has shown that two proteins containing this domain, LIC_11122 (Lsa19) and LIC_11834 (Lsa33) (Domingos et al., 2012; Figueredo et al., 2017), were able to actively interact with components of the human extracellular matrix and plasma *in vitro*. Furthermore, the lipoprotein LipL45 (LIC_11643), an abundant OMP that also possesses this domain, has previously been characterized as a candidate adhesin *in vitro* (Daroz et al., 2021). Moreover, previous studies with pathogenic *Leptospira* demonstrate that iron limitation acts as a potent environmental signal, inducing the bacterium to reprogram its overall metabolism and significantly alter the expression profile of its OMPs (Lo et al., 2010).

This work aims to functionally characterize two proteins with FecR domains from *L. interrogans* serovar Copenhageni, LIC_10902 and LIC_20185, previously predicted as hypothetical proteins. Additionally, LIC_10902 was divided into two fragments, F1 (with FecR domain) and F2 (without the FecR domain), to evaluate whether the FecR domain has a role in the interaction with host components.

## Materials and methods

### Animal and human ethics committee approvals

This study was performed accordingly to the guidelines outlined by the Brazilian National Council for Control of Animal Experimentation (CONCEA), which follows international guidelines for animal welfare and the principles of the 3Rs. Experimental protocols comply with the ARRIVE guidelines and were approved by the Ethic Committee on Animal Use of the Butantan Institute, São Paulo, Brazil (under the protocol number 3572060723). Mice were housed in a BSL1 animal facility, in micro isolators with individual ventilation and temperature and light cycle control. Animals received food and water *ad libitum* and manipulation was performed by trained personnel. Animals were euthanized by intraperitoneal injection of ketamine (300mg/kg) and xylazine (30mg/kg).

Human serum samples were obtained from the Adolfo Lutz Institute, São Paulo, Brazil, approved by the Ethics Committee (protocol no. 2.834.589). This study, jointly conducted by Instituto Butantan and Instituto Adolfo Lutz in São Paulo, received ethical clearance from the National Research Ethics Commission (CONEP) under CAAE protocol 78770117.0.0000.8098 via the Plataforma Brasil database.

### Bacterial strains and growth conditions

*E. coli* strains were cultured at 37 °C under aerobic conditions in BD Difco™ LB (Luria-Bertani) Broth Miller (#244620). For cloning of the target genes into the pAE vector, the *E. coli* DH5α strain was grown in LB medium supplemented with ampicillin (100 µg/mL) (Sigma-Aldrich, A9518). *E. coli* strain BL21 Star (DE3) pLysS (Invitrogen) and *E. coli* BL21 (DE3) were used for recombinant protein production. When cultured, *E. coli* strain BL21 Star (DE3) pLysS media was supplemented with 34 µg/mL of chloramphenicol (Sigma-Aldrich, C-0378) due to the maintenance of the pLysS plasmid. For cloning and conjugation between *Lb*P and *E. coli*, the auxotrophic *E. coli* strains π1 and β2163 were used, respectively. *E. coli* π1 was grown in LB medium supplemented with 40 µg/mL of spectinomycin (Sigma, S4014) and 0.3 mM of thymidine(Sigma, T1895), while *E. coli* β2163 was cultivated in LB supplemented with 40 µg/mL of spectinomycin and 0.3 mM of diaminopimelic acid (DAP) (Sigma, D4377). Additionally, *E. coli* MC1061 was used for the human serum resistance assays and was cultured in LB medium without antibiotics. Pathogenic *L. interrogans* serovar Copenhageni strains Fiocruz L1-130, and saprophytic *Lb*P were cultured at 30 °C under aerobic conditions in liquid Difco Ellinghausen–McCullough/Johnson–Harris (EMJH) basal medium (BD Difco, #279410) supplemented as previously described (Ellinghausen and McCullough, 1965; Johnson and Harris, 1967). Recombinant *Lb*P were cultured in EMJH medium, with the addition of 0.1 mg/mL of 5-fluorouracil (5-FU) (Sigma, F6627), to which leptospires are naturally resistant, and spectinomycin (40 µg/mL) for selection of the recombinant plasmid. Quantification of leptospires was performed by dark-field microscopy using a Petroff-Hausser counting chamber.

### *In silico* analysis

The genes encoding the proteins LIC_10902 and LIC_20185 proteins were obtained from National Center for Biotechnology Information database and subjected to analysis to predict protein conformation, domains, cellular localization, and signal peptide. ProtParam software (Gasteiger et al., 2005) was used to estimate the isoelectric point and molecular mass of both the wild-type and recombinant proteins. PFAM (Blum et al., 2025; Paysan-Lafosse et al., 2025) and SMART (Letunic and Bork, 2018; Letunic et al., 2021) were used to identify conserved domains. CELLO (Yu et al., 2004), PSORTb (Yu et al., 2010), TMHMM (Krogh et al., 2001), and LipoP 1.0 (Juncker et al., 2003) were employed for cellular localization predictions, while SignalP 6.0 (Petersen et al., 2011) was used to identify the signal peptide. WebCutter (Gagniuc et al., 2011) was used for restriction enzyme site predictions within the target gene sequences. Using the BLASTp tool from the National Center for Biotechnology Information (NCBI) and the Clustal Omega program (Madeira et al., 2022), a conservation tree was generated based on the amino acid sequence conservation of the target proteins across different *Leptospira* species.

### Cloning, production and purification of rLIC_10902 and rLIC_20185

To amplify the target genes from the *L. interrogans* serovar Copenhageni genomic DNA, primers were designed (**Table 1**) containing the *Bam*HI and *Nco*I restriction sites added to the 5’ ends of the oligonucleotides. Purified amplicons were digested to create cohesive ends for cloning in the pAE vector (Ramos et al., 2004). Chemically competent *E. coli* DH5α cells were used to amplify the recombinant plasmids. Colony PCR using T7 forward and pAER reverse primers (**Table 1**) was performed to identify positive clones. The PCR products were sequenced by the chain termination method (Sanger et al., 1977) on an ABI automated sequencer (PE Applied Biosystems). The resulting chromatograms were analyzed using SnapGene Viewer software (Version 5.1.7; GSL Biotech).

**Table 1:** List of primers used on the study.

Cloning in pAE vector			
Target protein	Primer	Restriction enzymes*	Nucleotide sequences (5’ - 3’)
LIC_**10902**	Forward	*Bam*HI	AATGGATCCGATTATACTCGATACGGA
	Reverse	*Nco*I	AAGCCATGGCATTCTCAAAAATTCTCT
LIC_**20185**	Forward	*Bam*HI	CCGCGGATCCTGTAAAGAAAATAAATCA
	Reverse	*Nco*I	GGCCCCATGGTTCAATAGATTCTAATTT
**F1**	Forward	*Bam*HI	AATGGATCCGATTATACTCGATACGGA
	Reverse	*Nco*I	AATCCATGGAGGCGACATGGGAAAAGG
**F2**	Forward	*Bam*HI	AATGGATCCAAAAAATCTTTGGACGGA
	Reverse	*Nco*I	AAGCCATGGCATTCTCAAAAATTCTCT
Cloning in pMG vector			
Target protein	Primer	Restriction enzymes*	Nucleotide sequences (5’ - 3’)
**LIC_10902**	Forward	*Nde*I	GGCCCGGCGCATATGAAAGATAAAATTCTTTCAGG
	Reverse	*Xba*I	GAGAGTCTAGATTA**GTGATGGTGATGGTGATG**CATTCTCAAAAATTCTCTTTGC
**LIC_20185**	Forward	*Nde*I	GCGCCGCCGCCATATGAAAAAAATATCTATATTATTTGTTTTAGTGG
	Reverse	*Xba*I	GAGAGTCTAGATTA**GTGATGGTGATGGTGATG**TTCAATAGATTCTAATTTCTTTTTTGC
Primers for Colony PCR			
Plasmids	Primer		Nucleotide sequences (5’ - 3’)
pAE plasmids	T7 Forward		TAATACGACTCACTATAGGG
	pAE Reverse		CAGCAGCCAACTCAGTTCCT
pMG plasmids	Forward		GCACTCTCACGAATATAGTGC
	Reverse		CCCATTTTCTGGAGTAGGTAGG

*Restriction cleavage sites are underlined; C-terminal his-tag sequence is highlighted in bold.

*E. coli* strain BL21 Star (DE3) pLysS was used for rLIC_10902, rLIC_20185 and F2 production F1 was produced by *E. coli* BL21 (DE3). Proteins production was carried out as described previously (Gaspar et al., 2024). rLIC_10902, rLIC_20185 and F2 were purified by metal affinity chromatography, as described (Gaspar et al., 2024), while F1 was purified by washing inclusion bodies (Silva et al., 2025). Concentration was estimated using the Bradford assay (Sigma-Aldrich, B6916). F1 and F2 final concentrations were determined by ImageJ densitometry. For both assays, bovine serum albumin (BSA) (A7906, Sigma-Aldrich) was used to generate a standard curve. Samples were subjected to three dialysis cycles against a 20 mM Tris and 500 mM NaCl buffer. Recombinant proteins were not tested for multimerization.

### Cloning and production of LIC_10902 and LIC_20185 in *Lb*P

For production in *Lb*P, expression cassettes were constructed using the pMaGroES (pMG) vector (Gaultney et al., 2020). These cassettes included the full-length coding sequences of LIC_10902 or LIC_20185. A 6x-histidine tag was added to the C-terminus of each protein sequence immediately before the stop codon. Additionally, *Nde*I and *Xba*I restriction sites were incorporated using the primers listed in **Table 1**. The target genes were amplified from *L. interrogans* sv. Copenhageni genome, purified and digested prior to cloning in the pMG vector. The recombinant vectors were amplified by *E. coli* π1 and verified by whole-plasmid sequencing (PLASMID-EZ, Genewiz/Azenta Life Sciences). Data analysis was performed using DNASTAR Lasergene 17 (SeqBuilder Pro and MegAlign Pro). Plasmids were used to transform electrocompetent *E. coli* β2163 cells and were subsequently transferred *Lb*P as previously described (Pappas et al., 2015; Fernandes et al., 2021; Surdel et al., 2022). *Lb*P transformed with the empty pMG vector (Gaultney et al., 2020; Surdel et al., 2022), was used as a negative control.

For analysis of FecR protein production in recombinant leptospires, cultures were collected, washed in PBS, and resuspended in 2x sample buffer (62.5 mM Tris–HCl pH 6.8, 10% glycerol, 2% SDS, protease inhibitor cocktail) at a 1:1 ratio to obtain a final concentration of 4 × 10^7^ cells/µL, and incubated at 100 °C for 10 min. The equivalent to 4 × 10^8^ cells/well were loaded onto 12.5% polyacrylamide gels. Protein extracts were separated by SDS-PAGE and analyzed by Coomassie staining and western blotting. FecR proteins were detected using a monoclonal anti-His6 IgG antibody (Invitrogen, MA1-135) diluted 1:1,000, followed by HRP-conjugated anti-mouse IgG antibodies (1:10,000) (Promega, W4021). Reactivity was detected using the Clarity™ Western ECL Substrate kit (Bio-Rad, #1705061).

### Production and adsorption of polyclonal sera against the recombinant proteins

To produce hyperimmune serum, BALB/c mice were immunized subcutaneously with 10 µg of rLIC_10902, F1, F2 or rLIC_20185 adsorbed onto 0.03% Al(OH)_3_, essentially as described (Gaspar et al., 2024). The pooled mice sera obtained through immunization were adsorbed against *E. coli* BL21 Star (DE3) pLysS antigens as described in the literature (Gruber and Zingales, 1995), in order to reduce cross-reactivity.

### Titration of hyperimmune mouse serum and detection of the target proteins

Serum titration was performed by ELISA using 96 well plates (High Binding, Costar, Corning Incorporated). Each well was incubated with 250 ng of recombinant protein for 16h at 4 °C. Plate wells were washed 3 times with PBS containing 0.05% Tween-20 (PBS-T) and then incubated with PBS-T containing 10% skim milk (PBS-T 10% milk) for 2h at 37 °C. Mouse serum was used as the primary antibody and diluted in PBS-T 10% milk. A serial dilution of the polyclonal serum was prepared, starting at a 1:100 ratio of serum to blocking solution. Primary antibodies were incubated for 1 h 30 min at 37 °C. After three washes with PBS-T, the plates were incubated with a horseradish peroxidase (HRP)-conjugated anti-mouse IgG secondary antibody (Sigma-Aldrich, A8667) for 1 h at 37 °C. The reaction was developed with a solution of O-phenylenediamine dihydrochloride (OPD) (1 mg/mL) (Sigma-Aldrich, P8287) and hydrogen peroxide (1 µL/mL) diluted in citrate-phosphate buffer (pH 5), and absorbance was measured at 492 nm using an ELISA reader (Multiskan-FC, Thermo Fisher Scientific).

SDS-PAGE gels were transferred onto 0.45 µm nitrocellulose membranes (Bio-Rad, #1704158) using the Trans-Blot Turbo Transfer System (Bio-Rad) for 7 min at 25 V and 1 A/cm² in Tris-Glycine transfer buffer (Bio-Rad, #10026938). Subsequently, the membrane was incubated in a blocking solution consisting of PBS-T 10% milk and incubated at 4 °C for 16 h, washed three times with PBS-T to remove the excess of unbound blocking proteins and incubated with hyperimmune mouse serum raised against the target proteins (1:5,000). After washing, hyperimmune serum was used as the primary antibody, a secondary HRP-conjugated anti-mouse IgG antibody (1:5,000) was added (Sigma-Aldrich, A8667), followed by 1 h of incubation at room temperature. Reactive bands were detected using the Amersham™ ECL Select™ Western Blotting Detection Reagent (GE Healthcare, #RPN2235).

### Tertiary structure evaluation

Circular dichroism spectra were recorded using a 1-mm path-length cuvette, at a scanning speed of 0.5 nm/s, on a Jasco J-810 spectropolarimeter (Japan Spectroscopic). Prior to the measurements, the samples were dialyzed against sodium phosphate buffer (0.1 M Na_2_HPO_4_ and 0.1 M NaH_2_PO_4_), pH 7.5, at 4 °C for 16 h. The spectra, expressed as mean residue molar ellipticity, were analyzed with the BeStSel server (Micsonai et al., 2018, 2022), which estimates protein secondary-structure content based on the experimental ellipticity values within the 185–250 nm absorption range. Complementarily, PSIPRED (Jones, 1999), AlphaFold (Jumper et al., 2021; Mirdita et al., 2022; Varadi et al., 2024) and PyMOL 2.5.4 (Student license) were used to analyze the three-dimensional structures of the proteins.

### Analysis of target proteins subcellular localization by fluorescence-activated cell sorting

Low passage *L. interrogans* serovar Copenhageni strain Fiocruz L1–130 cultures at the concentration of 10^8^ cells/mL were centrifuged (2,000 × g for 15 min), washed in 10 mL of Difco Dulbeccos PBS (DPBS) (Merck, #59331C) and resuspended in 200 uL PBS supplemented with 1% BSA. Mouse polyclonal serum against rLIC_10902, rLIC_20185, rLipl46 (outer membrane control), and rVapB (cytoplasmic control) (1:50 v/v in PBS- 1% BSA) were added to cell cultures, followed by incubation overnight at 30 °C. After washing, secondary FITC-conjugated anti-mouse IgG in PBS- 1% BSA (1:100 v/v) (Sigma-Aldrich, F9006) was added and cells incubated for 2 h at 30 °C. Cells were then washed and resuspended in PBS with 2% paraformaldehyde (Sigma-Aldrich, P6148). Measurement of fluorescence was performed in a BD FACSCanto II, and data are expressed as median fluorescence intensity (MFI) (Santos et al., 2018; Silva et al., 2025; Costa et al., 2026).

### Evaluation of the reactivity of recombinant proteins with leptospirosis serum samples by ELISA

500 ng/well of recombinant proteins were incubated on 96-well ELISA plates. Coating and blocking were performed as described previously. Leptospirosis serum samples were used as primary antibodies at 1:100 dilution in PBS-T 10% milk. Leptospirosis serum samples in the initial (MAT negative) and convalescent (MAT positive) phases of the disease were used. Recombinant proteins were incubated with serum samples for 1 h at 37 °C. Unbound primary antibodies were removed by an additional washing cycle with PBS-T. Detection of recombinant protein–serum interactions was performed using HRP-conjugated anti-human IgG secondary antibody (1:5,000) (Sigma-Aldrich, A8667) diluted in blocking solution, followed by 1 h of incubation at 37 °C. Excess secondary antibody was removed by two washing cycles with PBS-T. ELISA development was carried out using OPD and H_2_O_2_ as previously described. We only considered reactive values greater than three standard deviations above the mean of commercial NHS (Sigma-Aldrich, S1-MLSG). Our initial screening was performed using 100 MAT positive serum samples. If high reactivity were observed, 20 paired serum samples (MAT negative and MAT positive from the same patient) would be evaluated. Each serum sample was tested in duplicate, and at least two independent experiments were performed for each component group. The graphs shown correspond to a representative independent experiment.

### Interaction assay between recombinant proteins and host components

The interaction assay between the recombinant proteins and host plasma proteins was performed following the protocol previously described (Barbosa et al., 2006; Teixeira and Nascimento, 2020). Plasma fibronectin (human plasma, Sigma-Aldrich, F2006) (PF), VT (human plasma, Sigma-Aldrich, V8379), fibrinogen (human plasma, Sigma-Aldrich, F4883) (FB), PLG (human plasma, Sigma-Aldrich, P7999) were used, as well as complement proteins from Complement Technology, Inc: C3b (A114), C4b (A108), C4 binding protein (C4BP) (A109), C7 (A124), C8 (A125), and C9 (A126). All components were diluted in PBS and immobilized onto 96-well ELISA plates for 16 h at 4 °C. A total of 1 µg of each component was incubated per well, except for VT, for which 250 ng per well were used. As negative controls for interaction assays, BSA (Sigma-Aldrich, A7906), fetuin (Barbosa et al., 2006; Cameron et al., 2008) (fetal bovine serum, Sigma-Aldrich, F3385), and/or PBS-T containing 10% milk were included. Two independent experiments were performed for each component group and graphs shown correspond to representative results from two independent experiments.

### PLG capture assay directly from NHS

One microgram per well of rLIC_10902, rLIC_20185, or fetuin in PBS was added in 96-well ELISA plates. Washing, blocking, antibody detection and revelation were performed as described above. Commercial NHS 30% (Sigma-Aldrich, S1-MLSG) was serially diluted directly on the plate and incubated, allowing protein-PLG interaction. Primary antibody incubation was performed using anti-PLG antibodies (1:3,000) (Sigma-Aldrich, WH0005340), followed by detection with HRP-conjugated anti-mouse IgG antibodies (1:5,000) (Sigma-Aldrich, A8667).

### Evaluation of the effect of 6-aminocaproic acid on the interaction between recombinant proteins and PLG activation assay

PLG immobilization, washing, blocking, antibody detection and revelation were performed as described above. Recombinant proteins (1 µg/well) were then incubated in the absence or presence of ACA (2 or 20 mM) (Sigma-Aldrich, A2504). The plate was incubated for 2 h at 37 °C to allow PLG binding to the recombinant protein or to ACA. FecR protein immobilization, washing, blocking and incubation with purified PLG or NHS 30% were performed as described above. After protein-PLG interaction, the substrate D-Val-Leu-Lys p-nitroanilide dihydrochloride (Sigma-Aldrich, V0882) was added to a final concentration of 0.8 mM, along with urokinase-type plasminogen activator (uPA) (Sigma-Aldrich, U0633) to a final concentration of 0.1 ng/µL to convert PLG into PLA. The experiment was performed as described in (Vieira et al., 2009).

### Inhibition of C9 zinc-induced polymerization assay

The effect of recombinant proteins on the inhibition of C9 terminal complement polymerization was assessed. Different amounts of recombinant protein (1.25 μg, 2.5 μg, and 5 μg) were incubated with 3 μg of C9 in 20 mM Tris-HCl buffer (pH 7.4) for 40 min at room temperature. ZnCl_2_ (50 μM) was then added to induce C9 polymerization, followed by incubation for 2 h at 37 °C. Samples were separated on 4–20% gradient SDS-PAGE gels (Bio-Rad, #4561094) and stained with Coomassie Blue.

Positive controls for polymerization included samples containing only C9 with ZnCl_2_, and reactions containing an inert protein (5 μg fetuin), C9, and ZnCl_2_. Negative controls included purified C9 alone (no ZnCl_2_) and recombinant rLIC_13259 (5 μg) with C9 and ZnCl_2_. rLIC_13259 was previously described by our group as an inhibitor of ZnCl_2_-induced C9 polymerization (Cavenague et al., 2019).

Two independent experiments were performed for each FecR protein. The image shown corresponds to representative results from one independent experiment.

### NHS survival assay

Approximately 1,000 leptospires/mL were incubated either in EMJH medium supplemented with spectinomycin alone, medium containing 20% NHS, or medium containing 20% heat-inactivated normal human serum (iNHS). NHS was obtained from Innovative Research, Inc. (IPLA-SER-16728) and heat-inactivated at 56 °C for 30 min.

Bacterial survival was assessed after 1, 3, 6, and 24 h of incubation at room temperature. Leptospiral viability was determined by plating on LB agar followed by quantification of colony-forming units (CFUs). *E. coli* MC1061, a faster-growing control organism, was included to verify the bactericidal activity of the 20% NHS solution.

Leptospiral CFUs were counted 14 days after the end of the incubation period, whereas *E. coli* CFUs were counted after 1 day of incubation at 37 °C. Results were expressed as a survival index, calculated by dividing the number of CFUs obtained from the NHS or iNHS groups by the CFU count of the cultures incubated in medium alone at the corresponding time point. The experiment was performed four times with independent biological replicates.

### Statistical analysis

For the statistical evaluation of the ELISA-based assays assessing the interaction between target proteins and human components, the unpaired t-test was used.

Graphs were generated using GraphPad Prism 6 (GraphPad Prism, USA), and p values ≤ 0.05 were considered statistically significant. The dissociation constant (K_D_) was estimated by nonlinear regression performed in GraphPad Prism 6, using the “Binding–Saturation” model for a “One site – specific binding” interaction. NHS-resistance data assays were analyzed using GraphPad Prism 10 software by Kruskal–Wallis test. When significant, Dunn’s multiple-comparisons test was applied as a *post-hoc* analysis to determine which specific groups differed.

The workflow 1 summarizes all the steps performed.

## Results

### *In silico* analysis

ProtParam analysis estimated a molecular mass (MM) of approximately 42.5 kDa and an isoelectric point (pI) of 9.14 for rLIC_10902. For rLIC_20185, the MM was ≈ 28.2 kDa with a pI of 6.87. PFAM analysis revealed that both proteins contain a single domain, which correspond to a FecR domain of approximately 100 amino acids and is predicted to be exposed in the bacterial surface. Additionally, CELLO algorithms indicated a high probability of outer membrane anchoring for both proteins.

TMHMM analysis predicted LIC_10902 as a transmembrane protein, with the first 11 amino acids (aa) located intracellularly, residues 12–29 embedded in the membrane, and the sequence from the 30th aa onwards predicted as surface exposed. Conversely, LIC_20185 was identified as a putative lipoprotein by LipoP and SignalP. Both programs identified a signal peptide encompassing aa 1–19.

Conservation analysis showed that LIC_10902 and LIC_20185 (**Figures 1A, B**, respectively) are conserved among P1 subclade of *Leptospira* species capable of causing leptospirosis (Vincent et al., 2019).

**Figure 1:**
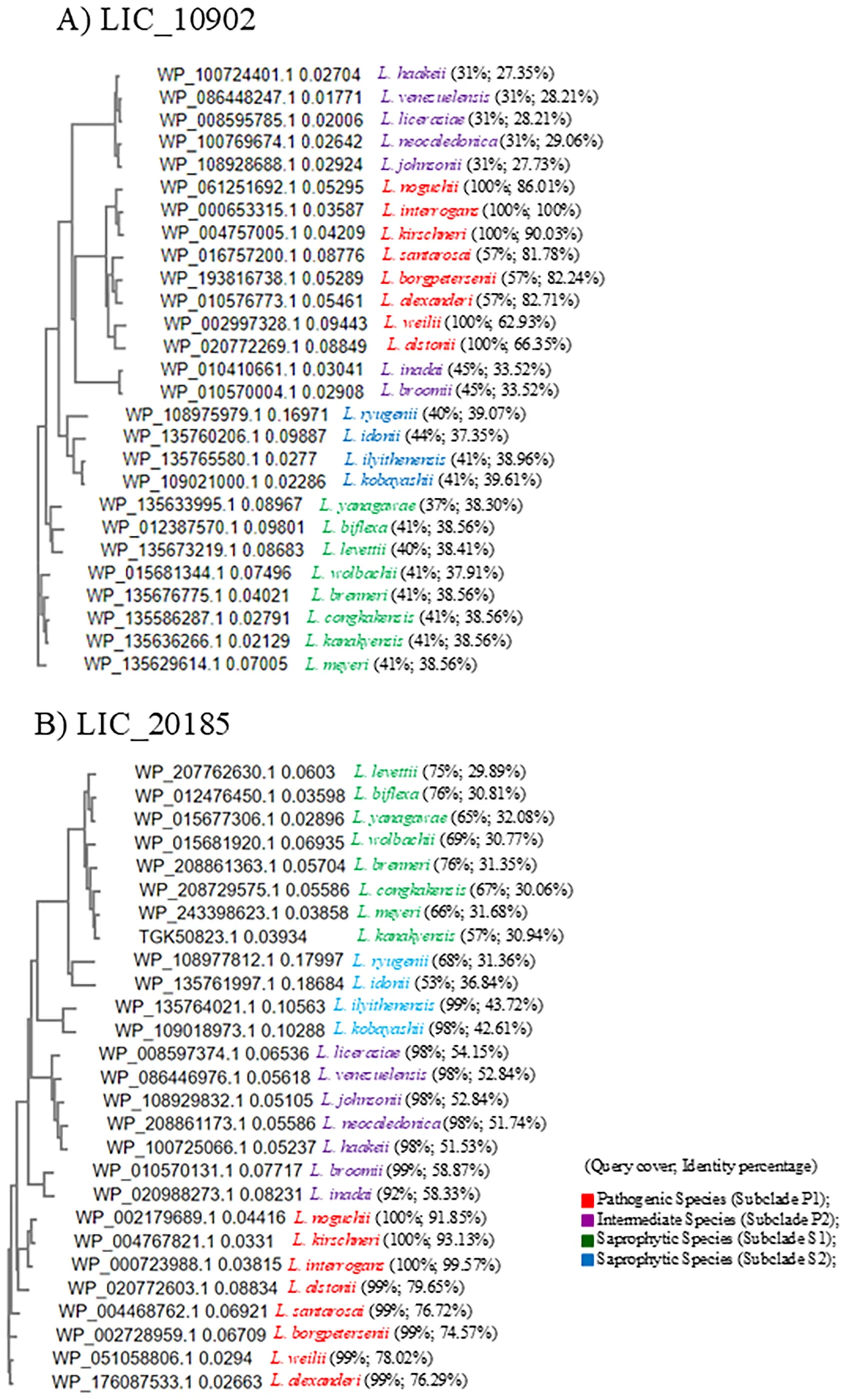
LIC_10902 and LIC_20185 conservation analysis among *Leptospira* genus and orthologs accession number. Phylograms were constructed using the Clustal Omega program based on sequence alignment data of the target proteins with different species of the genus *Leptospira*, performed by BLASTp. Values in parentheses represent: (percentage of sequence coverage by the database; percentage of identity). Colors divide the *Leptospira* species according to the taxonomy established by Vincent et al. (2019), where red represents pathogenic species, purple represents intermediate species, and blue and green represent saprophytes. **(A)** LIC_10902; **(B)** LIC_20185.

### Assessment of production and purification of recombinant proteins

After purification and dialysis, rLIC_10902, rLIC_20185 and fragments F1 and F2 were analyzed by SDS-PAGE (**Figures 2A, B**). The specific antibody titers in the pooled sera of animals immunized with rLIC_10902 or rLIC_20185 was 256,000 and 512,000. respectively. After four immunizations, the antibody titer of F1 or F2 immunized animals was 38,600 and 1,200, respectively. Titration of the pooled sera from the PBS control group confirmed the absence of specific antibodies against the target proteins throughout the experiment (not shown).

**Figure 2:**
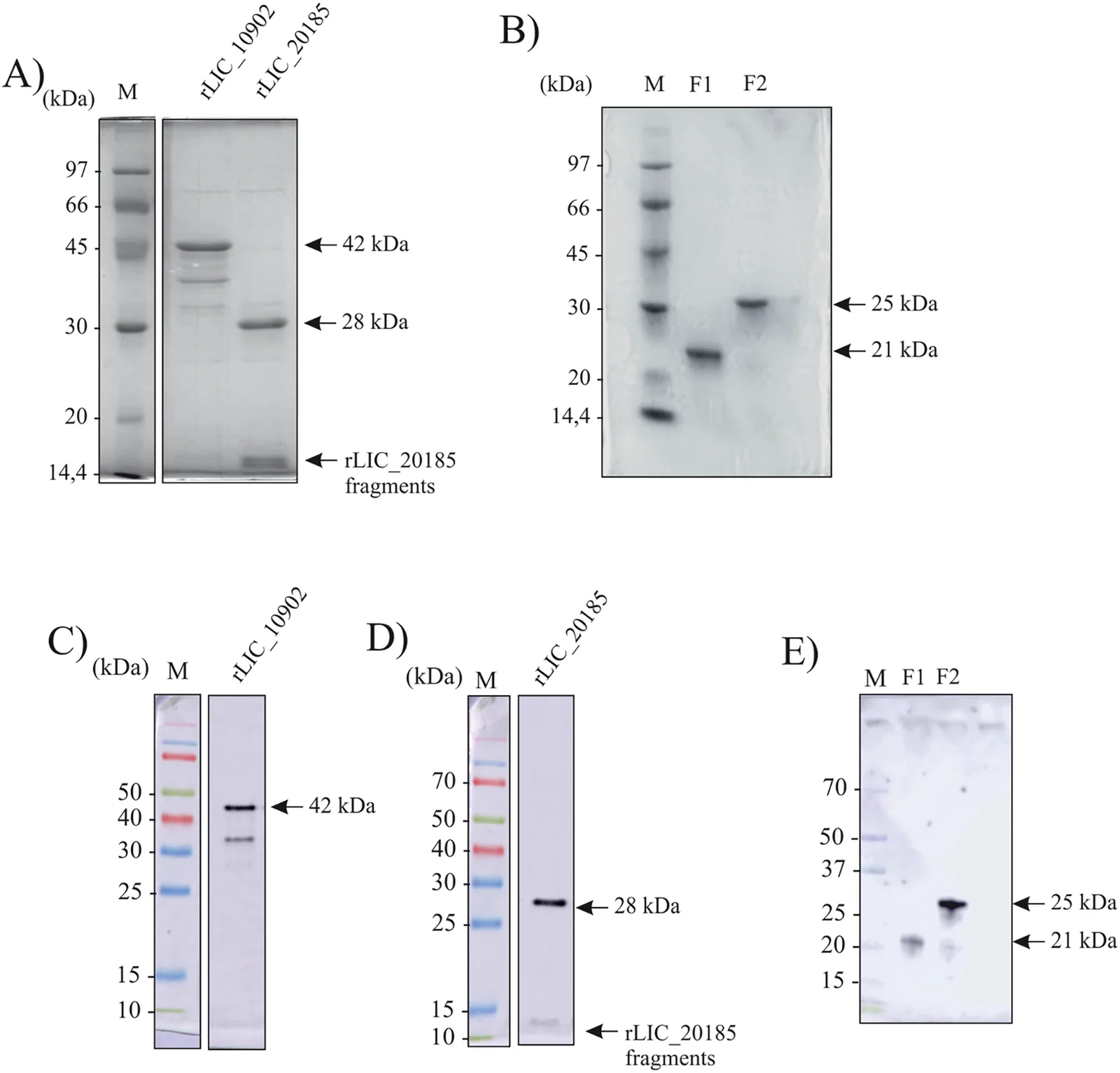
Purified protein analysis and detection by WB. A 12% polyacrylamide gel electrophoresis was performed, where only the molecular weight marker (M) and approximately 1 µg of **(A)** rLIC_10902 and rLIC_20185 or **(B)** F1 and F2 were loaded and gel was stained with Coomassie blue. After SDS-PAGE, approximately 50 ng of **(C)** rLIC_10902 or **(D)** rLIC_20185 or **(E)** 100 ng of F1 and F2 were loaded in the gel and transferred to a nitrocellulose membrane. The membrane was blocked and incubated with hyperimmune serum pool derived from the 3rd bleed (1:5,000). Subsequently, incubation was performed with peroxidase-conjugated anti-mouse IgG antibody (1:5,000) for later development. Arrows indicate the molecular mass range where the target proteins are located.

Pooled mouse sera detected the recombinant proteins by WB (**Figures 2C, D**). Interestingly, rLIC_20185 appeared to undergo fragmentation into two smaller products, ranging between 10 and 15 kDa. The fragment corresponding to the N-terminal region of rLIC_20185, which contains the 6xHis-tag, was also detected by the anti-polyhistidine antibody (data not shown). This specific characteristic was also further explored in the tertiary structure analysis section. F1 and F2 fragments were investigated for recognition by antiserum against rLIC_10902. Both fragments were identified by the anti-rLIC_10902 sera (**Figure 2E**). This result confirms the conservation of the rLIC_10902 epitopes in the F1 and F2.

### Subcellular localization analysis

FecR proteins subcellular localization was evaluated by FACS. **Figure 3**, shows the percentage of *Leptospira* labeling following incubation with different antibodies. Bacteria labeled only with FITC-conjugated IgG antibody (**Figure 3A**) were used to determine the threshold defining the percentage of bacteria specifically labeled with each antibody. It is observed that when the anti-VapB2 antibody was used as the primary antibody, followed by the addition of the FITC-conjugated secondary antibody, approximately 11.4% of the bacteria were labeled (**Figure 3B**). When the antibody produced against OMP LipL46 was used, 37.8% of the bacteria were labeled (**Figure 3C**). When anti-rLIC_10902 and anti-rLIC_20185 antibodies were used, approximately 52.9% and 77.7% of the bacteria were labeled (**Figures 3D, E**, respectively).

**Figure 3:**
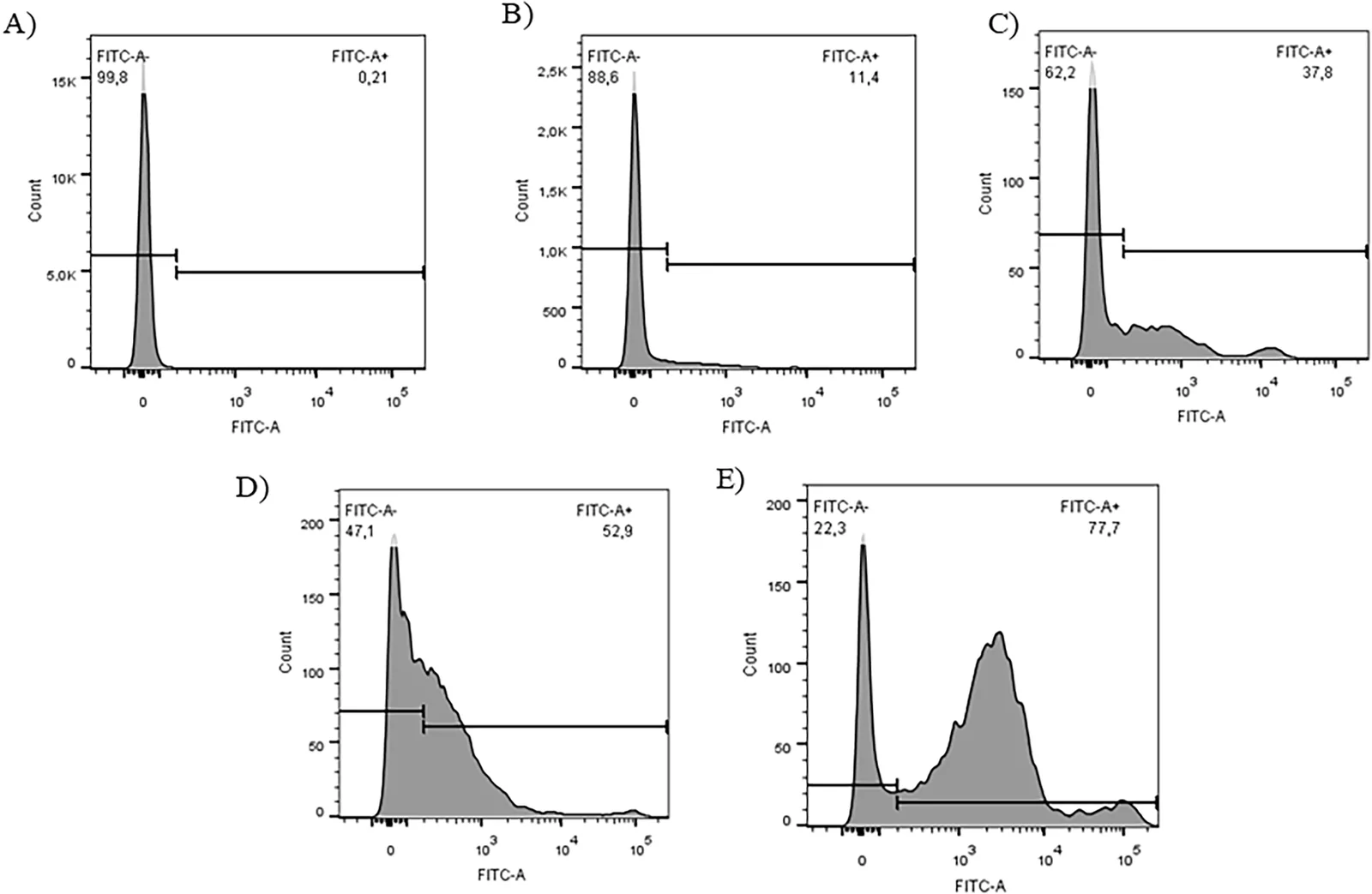
Subcellular localization assays. *L. interrogans* serovar Copenhageni strain L1–130 cells were analyzed by flow cytometry (FACS). **(A)** Leptospires labeled only with FITC-conjugated IgG (1:100); **(B)** bacteria incubated with mouse polyclonal anti-VapB2 serum (cytoplasmic protein); **(C)** anti-LipL46 (OMP); **(D)** anti-rLIC_10902; or **(E)** anti-rLIC_20185 (1:50), followed by the addition of FITC-conjugated anti-mouse IgG antibodies (1:100) to the reaction. Cells were recovered, washed, and the final pellet was resuspended in PBS solution with 2% paraformaldehyde. Samples were submitted to a BD FACSCanto II for fluorescence measurements. Horizontal bars define negative (left) and positive (right) FITC marked cell populations.

### Secondary and tertiary structure analysis

PSIPRED predictions suggested that the mature protein sequences possess a secondary structure, which was confirmed by CD (**Figures 4A, B**). The graphs reveal a positive peak around 185 nm and a corresponding negative peak in the 200 to 220 nm range, indicative of a predominantly β-sheet secondary structure (Johnson, 1988). Complementarily, BeStSel analysis predicted that the FecR domains are predominantly composed of β-sheets (**Table 2**). The presence of such secondary structure is an indicative of protein and FecR domain function.

**Figure 4:**
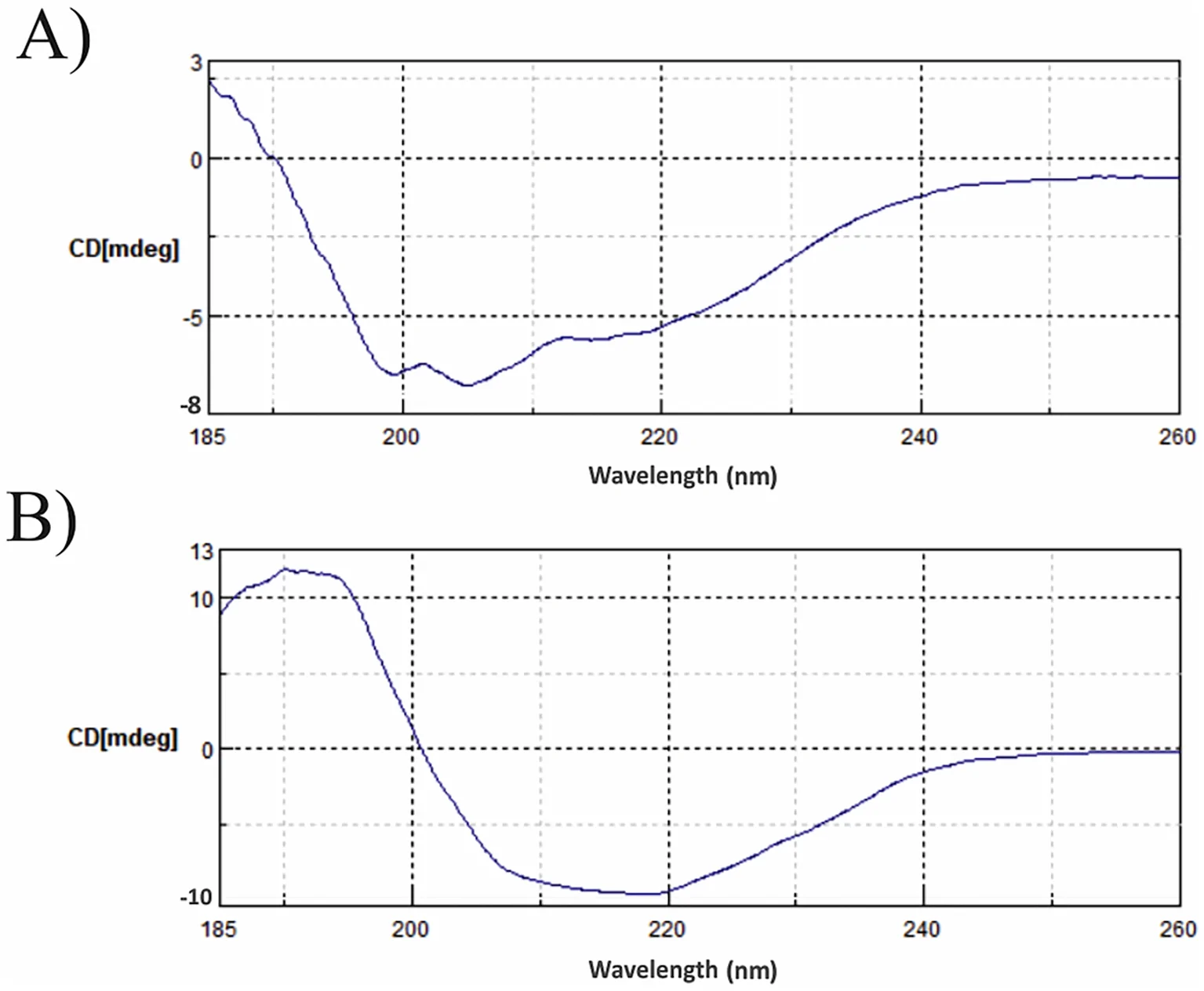
Recombinant proteins’ CD spectra. Following dialysis against 10 mM sodium phosphate buffer, pH 8.0, **(A)** rLIC_10902 and **(B)** rLIC_20185 were analyzed using a spectropolarimeter with a 1 mm optical path length cuvette at 0.5 nm/s intervals. Based on the graphs, a peak of positive signals is observed near 185 nm, along with a peak of negative signals between 200 and 220 nm.

**Table 2:** Secondary structure composition of the target proteins determined by PSIPRED and BeStSel (CD).

Recombinant proteins	Secondary structure	PSIPRED	BeStSel
rLIC_10902	α-helix	16%	5.5%
	β-sheets	28.2%	47.3%
	Irregular structure	55.8%	47.2%
rLIC_20185	α-helix	8.8%	9.3%
	β-sheets	48.1%	45.3%
	Irregular structure	43.1%	45.4%

Secondary structure percentages were predicted for both recombinant proteins. For rLIC_10902, the BeStSel analysis demonstrated a higher proportion of β-sheets and a lower contribution of α-helices and irregular structures compared to the PSIPRED prediction. Regarding rLIC_20185, the prediction was nearly identical to the experimental data. Both proteins exhibited a similar structural pattern, with α-helices accounting for less than 10% of the protein structure, while β-sheets and irregular structures contributed almost equally, each representing more than 45% of the total composition. These findings corroborate with AlphaFold tertiary structure modeling, visualized with PyMOL (**Figures 5A–D**). Additionally, **Figure 5** explores the similarities between LIC_20185 and LipL45 (**Figures 5E, F**). Both are FecR proteins that might undergo a similar autocleavage process mediated by a non-enzymatic interaction between two amino acids present in the GT motif (**Figures 5G, H**).

**Figure 5:**
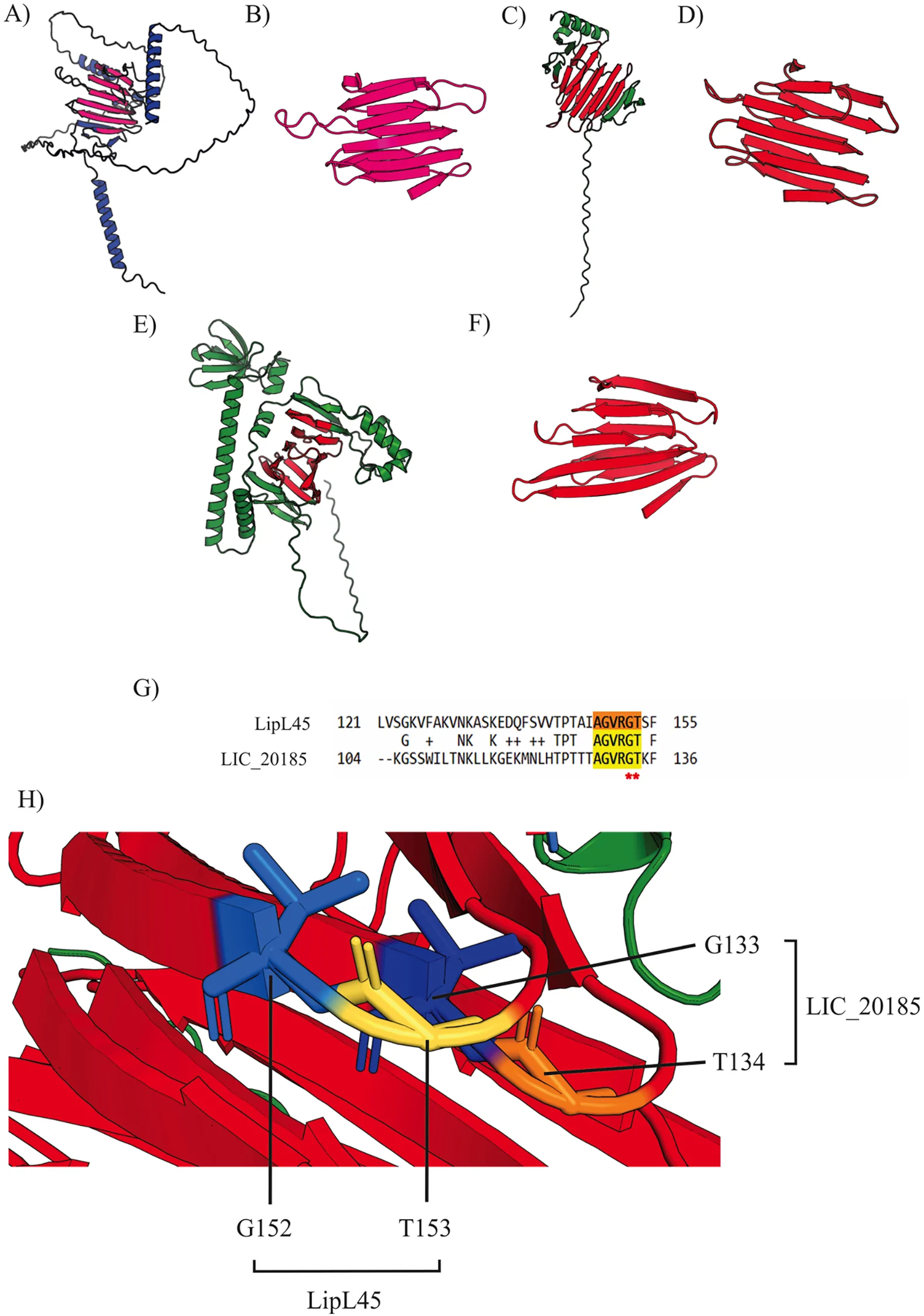
Evaluation of *L. interrogans* FecR protein’s tertiary structure and LIC_20185 fragmentation. The wild-type protein structures were imported from AlphaFold into PyMOL to highlight the FecR domains within the protein structure. **(A)** LIC_10902, with its FecR domain (76–174 aa) highlighted in magenta; **(B)** magnified view of the LIC_10902 FecR domain; **(C)** LIC_20185, with its FecR domain (60–155 aa) highlighted in red; **(D)** magnified view of the LIC_20185 FecR domain. **(E)** LipL45, with its FecR domain (74–175 aa) highlighted in red; **(F)** magnified view of LipL45 FecR domain. It is interesting to note that β-sheets are the primary structural component of the FecR domain. **(G)** Amino acid sequence alignment of the FecR proteins LipL45 and LIC_20185 from *L. interrogans* using the NCBI BLASTp tool, demonstrating the conservation of the GT motif. The G and T residues, which are conserved and represent the putative cleavage site, are indicated with red asterisks. The regions underlined in orange or yellow indicate positions with a single fully conserved residue. **(H)** Cartoon representation of the structural alignment between the LipL45 model and LIC_20185, magnifying the GT motif. **(A–F, H)** were prepared using PyMOL software.

### Evaluation of the reactivity of recombinant proteins with leptospirosis serum samples by ELISA

Initially, the reactivity of the recombinant proteins was evaluated against 100 serum samples from patients in the convalescent stage of leptospirosis (MAT positive), characterized by seroconversion and the presence of agglutinating antibodies against the pathogen. rLIC_10902 was reactive with 48% of the samples, while rLIC_20185 reacted with only 30%. Due to its reactivity with nearly 50% of the samples, rLIC_10902 was selected for further evaluation against 20 paired serum samples. The analysis revealed that rLIC_10902 was recognized by only 6.25% of the MAT negative sera and 50% of the MAT positive sera from the paired samples (not shown), suggesting that antibodies against LIC_10902 and LIC_20185 are produced later, most probably, during the convalescent phase of the disease.

### Interaction assay between recombinant proteins and host components

Since pathogenic *Leptospira* likely need to interact with the host ECM and cellular receptors to adhere to the host and start the infection process, we tested the FecR protein ability to bind different types of collagens, elastin, E-cadherin and laminin. rLIC_10902 did not interact with any of the ECM and E-cadherin molecules tested in this group (data not shown). However, rLIC_20185 interacted with ECM proteins and E-cadherin. As shown in **Figure 6A**, rLIC_20185 was able to bind to elastin, E-cadherin, and laminin; Only the interactions with elastin and E-cadherin were dose-dependent and saturable, thus considered specific (**Supplementary Figure 1**).

**Figure 6:**
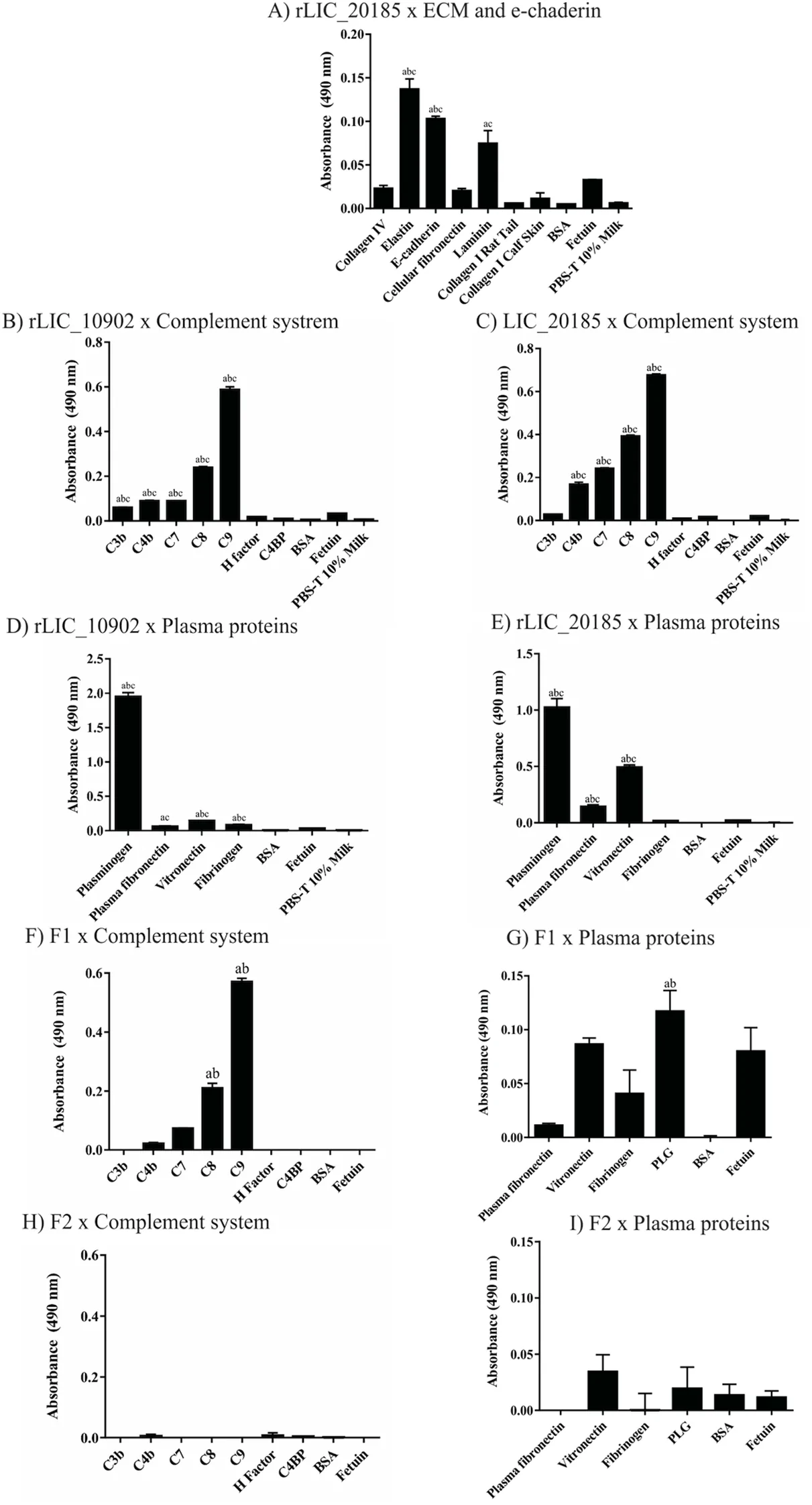
FecR proteins binding to host components evaluation by ELISA. Interaction of 1 µg of FecR proteins with host-components. Detection was carried out using mouse hyperimmune serum (1:5,000), followed by incubation with peroxidase-conjugated anti-mouse IgG antibody (1:5,000). Experiments were performed in duplicates, and the one-way ANOVA test was used for statistical analysis. Results are expressed as the mean ± standard deviation of a triplicate for the binding assay. Differences were considered statistically significant at p ≤ 0.05. Significant values are denoted by “a” (significance relative to BSA), “b” (relative to fetuin), or “c” (relative to 10% milk PBS-T). **(A)** Interaction between rLIC_20185 and ECM molecules and E-cadherin; Interaction between complement system proteins and **(B)** rLIC_10902 and **(C)** rLIC_20185; Interaction between plasma proteins and **(D)** rLIC_10902 and **(E)** rLIC_20185; Interaction between F1 and **(F)** complement system proteins and **(G)** plasma proteins; Interaction between F2 and **(H)** complement system proteins and **(I)** plasma proteins.

Additionally, considering the importance of complement resistance for the survival and maintenance of the pathogenic *Leptospira* life cycle, the capability of these proteins to interact with the following complement components was assessed: C3b, C4b, C4BP, C7, C8, and C9. Among the tested complement system components (**Figures 6B, C**), both proteins were able to bind to C4b, C7, C8, and C9 in a dose-responsive manner. Although interaction between rLIC_20185 and C3b was observed, it did not show a dose-responsive behavior.

In the host bloodstream, pathogenic *Leptospira* also interact with other key plasma proteins, such as PF, FB, VT, and PLG. Therefore, the interaction of FecR proteins to these components was also analyzed. The evaluation of interaction with other plasma proteins demonstrated that rLIC_10902 interacts *in vitro* with PLG, FP, and VT (**Figure 6D**). Similarly, rLIC_20185 interacted with PLG, VT, FP, and FB (**Figure 6E**). The interactions of both proteins with FP, and of rLIC_20185 with FB, did not show a dose-dependent pattern and were considered non-specific (not shown). Conversely, the interaction of both proteins to PLG and VT exhibited dose-dependent and saturable patterns (**Supplementary Figure 2**).

Also, we were able to demonstrate that the FecR domain (F1) mediates rLIC_10902 interaction with C8 and C9 (**Figure 6F**), and with PLG (**Figure 6G**). As can be seen, F2 could not interact significantly with any of the molecules analyzed (**Figures 6H, I**).

For dose-responsive interactions, the dissociation constant (K_D_) was calculated and displayed in **Table 3** below.

**Table 3:** K_D_ calculated from dose-response assays between the target proteins and host components (nM).

FecR protein/host component	rLIC_10902	rLIC_20185
E-cadherin	NR	881.70 ± 247.50
Elastin	NR	358.30 ± 74.14
C4b	658.1 ± 186.1	1444 ± 86.14
C7	622.7 ± 143.5	1633 ± 148.7
C8	739.7 ± 80.21	768.8 ± 82.94
C9	94.11 ± 10.89	126.5 ± 23.75
PLG	8.061 ± 0.5153	92.28 ± 7.21
VT	725.1 ± 40.19	480.7 ± 82

NR, not reactive.

### Plasminogen interaction and activation

PLG is a protein characterized by five kringle domains, which mediate its interaction with various ligands, including bacterial proteins. These domains typically interact with lysine residues present in the ligands (Castellino and Ploplis, 2005). As shown in **Figure 7A**, both rLIC_10902 and rLIC_20185 interact with the kringle domains of PLG via their lysine residues, as the interaction was reduced in the presence of varying concentrations of the lysine analogue, ACA. Complementarily, **Figures 7B, C** demonstrate that both proteins are capable of capturing PLG directly from NHS. Furthermore, when provided with either purified PLG (**Figure 7D**) or PLG derived from NHS (**Figure 7E**), PLG bound to proteins are converted in its active form, PLA, in the presence of uPA. Thus, it is possible that these proteins facilitate the leptospiral penetration through the host barriers by generation of proteolytic PLA.

**Figure 7:**
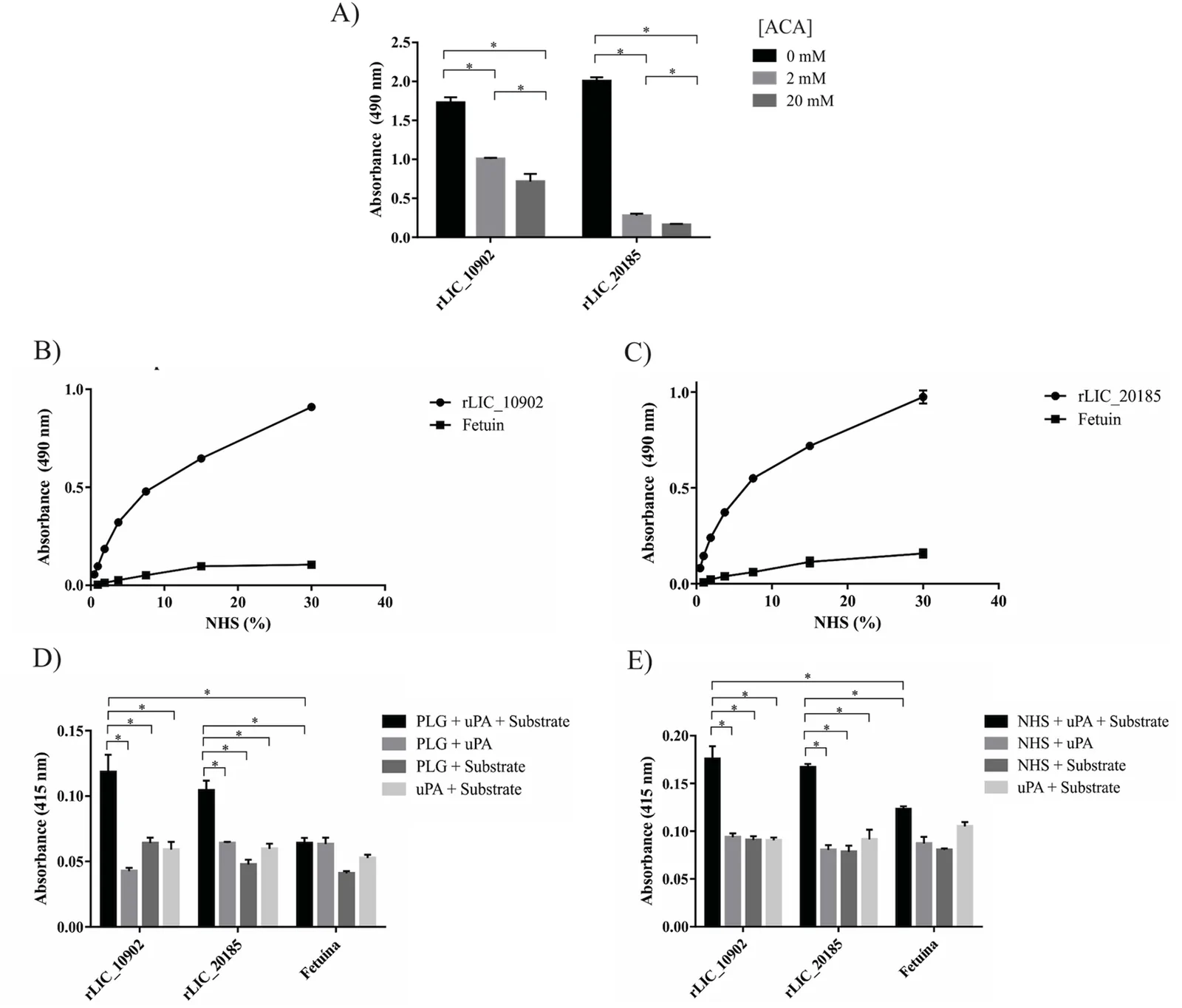
Characterization of the FecR proteins interaction with PLG by ELISA. **(A)** Evaluation of the effect of ACA (lysine analogue) on the PLG-recombinant protein interaction: One µg/well of PLG was immobilized on an ELISA plate. Subsequently, incubation of rLIC_10902 or rLIC_20185 was performed in the absence and presence of ACA at different concentrations (2 and 20 mM). Detection of the binding was performed using mouse hyperimmune serum (1:5,000) followed by detection with peroxidase-conjugated anti-mouse IgG antibody (1:5,000). Each column on the graph represents the mean of a triplicate and standard deviation. For each protein, all groups were compared among themselves, using the t-test and considering the difference between experimental groups significant for p < 0.05 (“*”); **(B, C)** PLG capture from NHS by FecR proteins assay: The proteins were immobilized on ELISA plates (1 µg/well) and subsequently incubated with 10% milk PBS-T blocking solution containing 30% NHS. Serial dilution (1:1) of the NHS in 10% milk PBS-T solution was performed for gradual reduction of the NHS percentage. Detection of PLG captured by the FecR proteins was performed with anti-PLG antibody produced in mice (1:5,000), followed by incubation with peroxidase-conjugated anti-mouse IgG antibody (1:5,000). Each point on the graph is represented by the mean ± standard deviation of a triplicate. **(D, E)** evaluation of the activation capacity of purified PLG **(D)** or PLG captured from NHS **(E)** in the presence of uPA by FecR proteins. After immobilization of the recombinant protein or fetuin (1 µg/well), there was incubation with 1 µg of PLG or 30% NHS in blocking solution, 0.8 mM uPA, and 0.1 ng/µL substrate (complete reaction), or PLG/30% NHS and uPA, or PLG/30% NHS and substrate, or uPA and substrate, forming a group in which all components were present and others in which one component was absent (negative controls). The reading is performed through the degradation of the substrate by PLA, responsible for creating a chromophore compound capable of being detected through wavelength reading at 415 nm. Each column on the graph represents the mean of a triplicate ± standard deviation. For each FecR protein, the value of the complete reaction was compared with the negative controls and with the complete reaction of the negative control protein, fetuin. The t-test was used and the difference between the experimental groups was considered significant for p < 0.05 (“*”).

### Inhibition of C9 zinc-induced polymerization assay

Given the importance of C9 component interaction for survival against complement system attack, the ability of FecR proteins to inhibit Zn^2+^-induced C9 polymerization was evaluated (**Figure 8**). Both FecR proteins were found to inhibit the polymerization of 3 µg (281.69 nM) of C9 in a dose-dependent manner. In **Figures 8A, B**, the first four lanes represent positive and negative controls. Lane 1 shows monomeric C9 (71 kDa), which does not polymerize in the absence of the inducer, ZnCl_2_. Lane 2 shows C9 after the addition of 50 µM ZnCl_2_, evidenced by a high-molecular-mass band above 250 kDa and the presence of residual monomeric C9. Lane 3 represents fetuin, a protein that does not inhibit C9 polymerization, while Lane 4 displays rLIC_13259, a protein known to inhibit this process (Cavenague et al., 2019). Lanes 5, 6, and 7 demonstrate that the inhibition of C9 polymerization is dependent on the concentration of the FecR protein. It was observed that 1.25 µg of rLIC_10902 (196.23 nM) or rLIC_20185 (295.84 nM) (Lanes 5) had no visible effect on polymerization. In contrast, 2.5 µg of rLIC_10902 (392.45 nM) or rLIC_20185 (591.67 nM) (Lanes 6) partially reduced the presence of C9 polymers. Moreover, 5 µg of rLIC_10902 (784.91 nM) or rLIC_20185 (1180 nM) (Lanes 7) completely inhibited polymerization, as evidenced by the total absence of C9 polymers and a corresponding increase in the monomeric C9 band. The data suggest that both proteins could interfere in MAC formation by inhibiting C9 polymerization.

**Figure 8:**
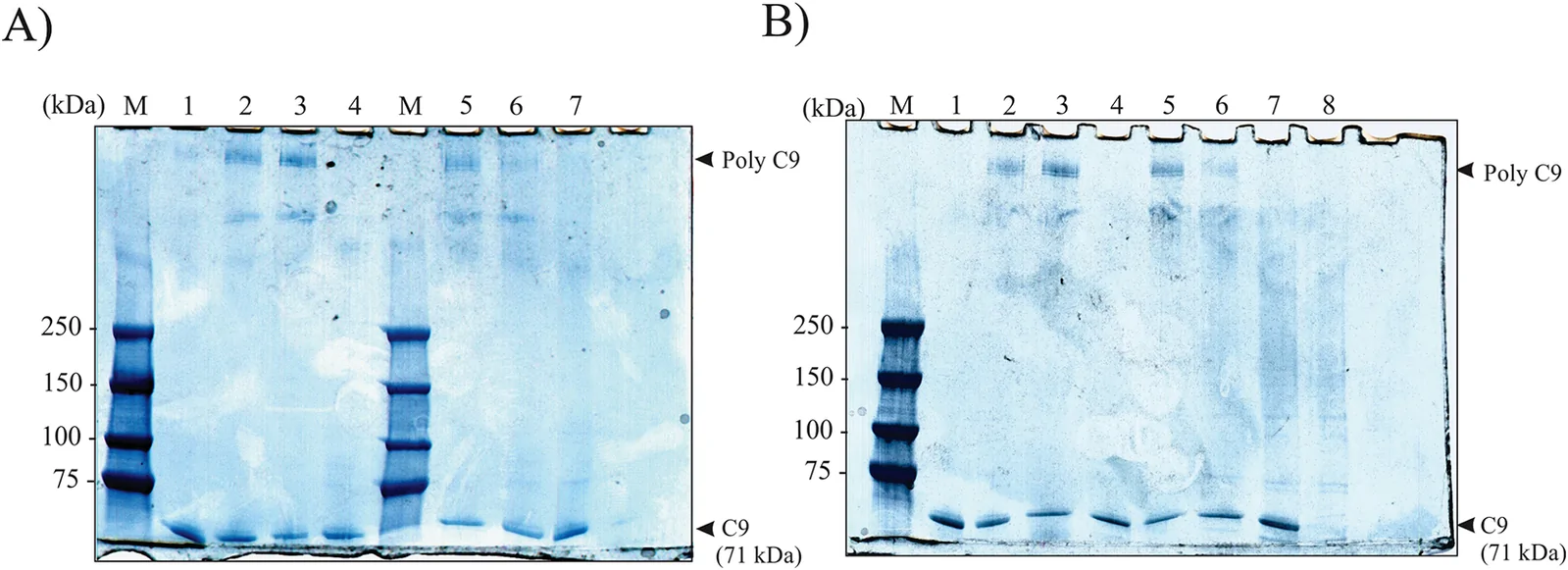
C9 polymerization inhibition assay by rLIC_10902 **(A)** and rLIC_20185 **(B)** proteins. Three µg of C9 were incubated in Tris-HCl buffer pH 7.2 for 40 min at 37 °C in the absence or presence of the tested proteins. Subsequently, C9 polymerization was induced for 2 h at 37 °C with 50 µM of ZnCl_2_. Samples were analyzed by polyacrylamide gradient gel electrophoresis (4-20%) with subsequent Coomassie blue staining. (1) C9 incubated in buffer without ZnCl_2_ and without other proteins, representing the negative control for C9 polymerization; (2) C9 incubated only with ZnCl_2_, representing a positive control for C9 polymerization; (3) C9 incubated with ZnCl_2_ and 5 µg of fetuin, acting as a negative control for the inhibition of C9 polymerization; (4) C9 incubated with ZnCl2 and 5 µg of rLIC_13259, acting as a positive control for the inhibition of C9 polymerization; (5 – 7) C9 incubated with ZnCl_2_ and 1.25, 2.5, or 5 µg of FecR proteins, respectively. In columns 1, 4, and 7, the absence of polymerized C9 is noted, resulting in an increase of monomeric C9.

### Production of the FecR proteins by saprophytic *Leptospira*

The recombinant pMG plasmids, containing the coding sequences for each FecR protein, were used to transform *E. coli* β2163. This donor strain was subsequently employed for conjugation with wild-type *Lb*P. After a two-week incubation period, recombinant leptospiral colonies were recovered. Transformed clones, pMG. LIC_10902.6xhis and pMG. LIC_20185.6xhis were maintained for two to three passages (approximately two to three weeks) in supplemented EMJH. To determine whether the FecR proteins were produced by *Lb*P pMG.LIC_10902.6xhis and *LbP* pMG.LIC_20185.6xhis, bacterial extracts from four clones of each plasmid construct were analyzed. Samples from *LbP* pMG (wild-type *Lb*P transformed with the empty vector) and the purified recombinant FecR proteins were included as controls.

Although the target proteins could not be detected in the leptospiral extracts through Coomassie Blue staining (data not shown), WB analysis confirmed that all clones were producing the target proteins **Figures 9A, B**, suggesting that although expression was successful, the recombinant bacteria did not produce high levels of the protein.

**Figure 9:**
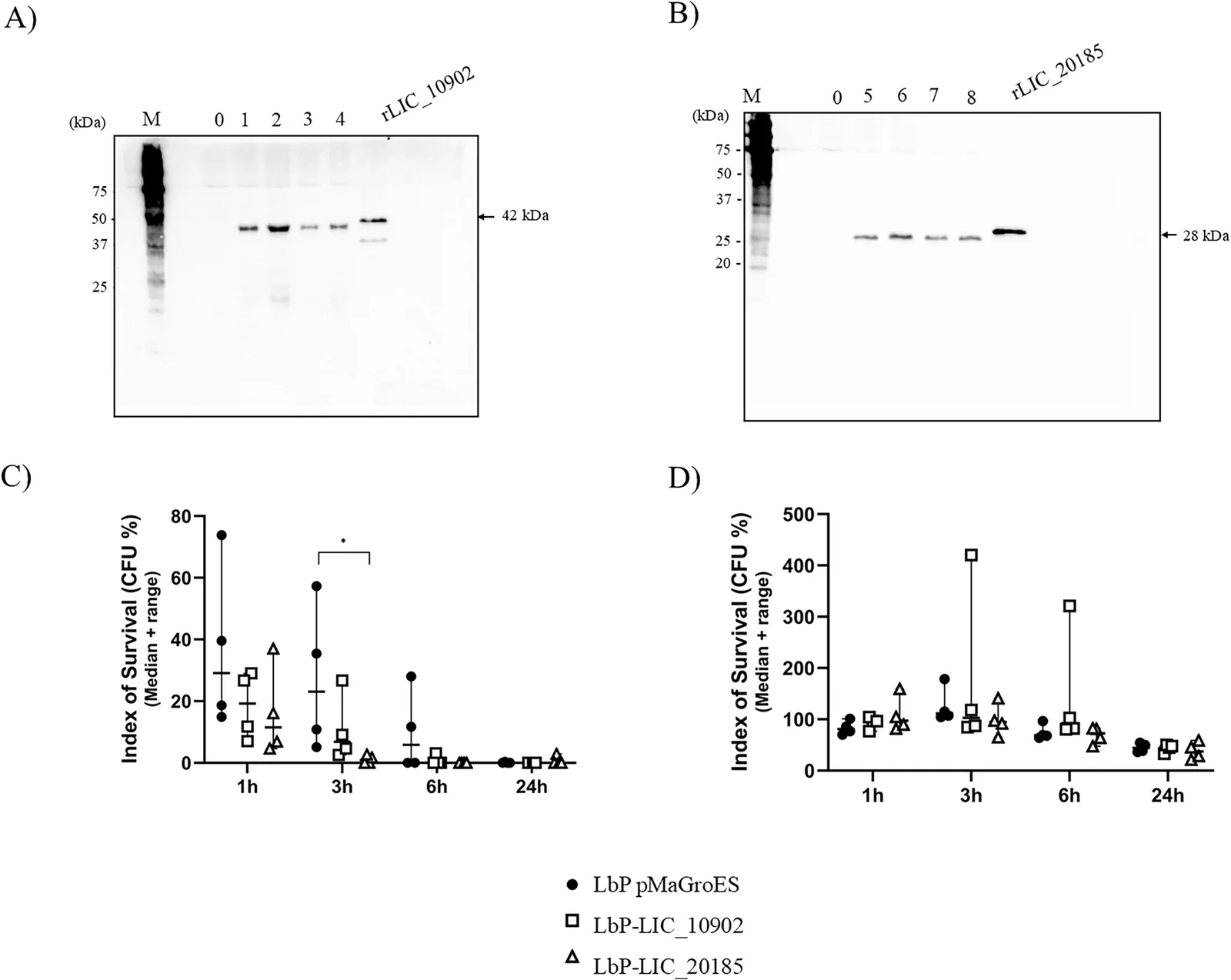
Analysis of heterologous FecR protein production and survival of recombinant *Leptospira* CFUs after 1, 3, 6, and 24 h of incubation with NHS and iNHS. The production of recombinant proteins by *Lb*P was evaluated by immunoblotting **(A)** LIC_10902 production and **(B)** LIC_20185 production. Approximately 4 x 10^8^ bacteria were applied per well. (0): *Lb*P-pMG; (1,2,3 and 4): *Lb*P-pMG.LIC_10902.6xhis clones; (5, 6, 7 and 8): *Lb*P-pMG.LIC_20185.6xhis clones. **(C, D)** The *Lb*P strains, diluted to an approximate concentration of 1,000 cells/mL, were incubated in EMJH containing 20% NHS **(C)** or 20% iNHS **(D)** for different periods. CFUs were quantified 14 days after the end of the incubation. Data represents the group’s median and range. *Lb*P*-*pMG.LIC_10902.6xhis and *Lb*P-pMG.LIC_20185.6xhis were compared with *Lb*P-pMG using Kruskal-Wallis tests and Dunn’s multiple comparisons. “*” indicates p ≤ 0.05.

### NHS survival assay

To survive within the host serum, virulent leptospires must be resistant to the complement system and capable of evading other immune components (Vieira et al., 2014). Given that rLIC_10902 and rLIC_20185 interact with high affinity with recombinant human C9 and inhibit its polymerization *in vitro*, whether the expression of these FecR proteins would confer resistance to human complement attack in *LbP* pMG. LIC_10902.6xhis and *LbP* pMG. LIC_20185.6xhis strains were evaluated. **Figure 9C** displays the survival rate (CFU counts) after incubation with 20% active NHS. As observed, the production of FecR proteins did not result in increased survival of the recombinant leptospires. On the contrary, the production of FecR proteins negatively affected the survival of *LbP* pMG. LIC_20185.6xhis at the 3-hour time point.

The survival of the three *Lb*P derivatives was not significantly different after exposure to heat-inactivated sera (**Figure 9D**), and it is worth noting that the NHS effectively inhibited *E. coli* growth, confirming its bactericidal activity (not shown).

## Discussion

In this study, it is reported that LIC_10902 and LIC_20185, two FecR domain-containing proteins from *L. interrogans*, are at least partially located in the leptospiral outer membrane and interact with several host components, more specifically with PLG and C9, which seems to be mediated by the FecR domain. Both proteins are conserved among pathogenic *Leptospira* species, and their coding sequences. Together, our results may indicate that these proteins may play a possible role in bacterial pathogenesis and virulence.

Accordingly, to the predictions, LIC_10902 possesses a similar localization and structure as *E. coli* FecR protein, holding a cytoplasmatic and transmembrane N-terminal portions, while the rest of the protein, including the FecR domain, would be exposed outside de membrane. LIC_20185 is a putative lipoprotein. Both are predicted to be, at least partially, exposed in the bacterial outer membrane, which was corroborated by FACS evaluation (see **Figure 3**). The percentage of cells labeled with the anti-LipL46 antibody for FACS is well-established in the *Leptospira* field, and the results obtained in this experiment corroborate published data (Santos et al., 2018; Silva et al., 2025). The fact that anti-FecR protein antibodies were able to label an even higher percentage of cells than the anti-LipL46 antibody is a strong indicator that both proteins are located, at least partially, on the outer membrane of the *L. interrogans*.

Conservation analysis indicated that LIC_10902 is highly conserved among P1 subclade, while LIC_20185, presented a high coverage rate across P1 and P2 subclades. This feature might be influenced by the protein size (233 aa). Overall, the identity between LIC_20185 residues and its orthologs is notably higher among pathogens, exceeding 50% identity between pathogenic and intermediate species, suggesting that these proteins could be important for bacterial virulence.

Interestingly, rLIC_20185 fragmented into two smaller products, ranging between 10 and 15 kDa (see **Figure 3**). Similar to *L. interrogans* LipL45 and *P. aeruginosa* FoxR, both LIC_20185 and its recombinant form (rLIC_20185) possess the conserved GT motif located in a loop region between two β-sheets that comprise the FecR domain (see **Figure 6**), indicating that may share the same self-cleavage mechanism, which is usually associated cell-surface signal transduction (Bastiaansen et al., 2015a, 2015b; de Assis Noman et al., 2024).

Both proteins were recognized by leptospirosis serum samples, indicating their production during infection. Nevertheless, neither FecR protein tested exhibited a relevant diagnostic potential, as shown by a chimeric protein that reacted against 75% of serum samples at the early stage of leptospirosis (Fernandes et al., 2022). These proteins, however, did raise antibody responses in mice, so are antigenic when administered with adjuvant. Together these data suggest that the proteins are not produced in the majority of human infections. During the infectious process, pathogenic leptospires utilize their OMPs to interact with host components throughout the infection. These OMPs are essential for adhesion, invasion, immune system evasion, and colonization of target tissues (Bharti et al., 2003). In this study, the FecR proteins and FecR-containing fragments of LIC_10902 were demonstrated to interact in a dose-responsive manner with a broad range of ligands.

Among ECM and other proteins that would be exposed during skin trauma, rLIC_20185 was able to interact with E-cadherin and elastin. Regarding the interaction with E-cadherin, the affinity of rLIC_20185 was similar to the rLIC_10831 protein dimer, which exhibited an affinity of 800 nM for this molecule (Eshghi et al., 2019). Additionally, other *L. interrogans* proteins, such as LipL21 (Takahashi et al., 2021) and OmpL37 (Pinne et al., 2010; Oliveira et al., 2011) have already been reported as elastin ligands. After adhesion, pathogenic leptospires must enter the blood vessels to be transported to the target organs (Ko et al., 2009; Cinco, 2010). Once in the bloodstream, the pathogen is challenged by the complement system and can interact with various plasma proteins. As the first line of host’s immune defense, the complement system proteins aim for pathogens membrane lysis by MAC polymerization (Sarma and Ward, 2011). In order to survive the passage into the host’s serum, pathogenic leptospires must be resistant to the complement system (Adler, 2014). Many *L. interrogans* proteins have been previously characterized as ligands of complement system components. The interaction of FecR proteins with components C4b, C7, C8, and C9 occurred in the same order of magnitude as the proteins rLIC_12587 (Kochi et al., 2019), rLIC_13259 (Cavenague et al., 2019), rLIC_10774 (Passalia et al., 2020), and rLIC_13086 (Passalia et al., 2021). In comparison with the *L. interrogans* OMPs that interact to the complement system, and the list of OMPs available in the **Supplementary Material** of Daroz et al. (2021), it is noteworthy that the proteins rLIC_10902 and rLIC_20185 exhibit the highest affinity for the terminal component C9, indicating that this interaction may occur under physiological conditions. In addition, the interaction of rLIC_10902 with C8 and C9 strongly suggests being mediated by its FecR domain, highlighting the role of this domain during infection.

It was observed that both FecR proteins are capable of inhibiting the zinc-induced polymerization of C9 in a dose-dependent manner, corroborating the results of the dose-dependency assays and suggesting that this interaction could occur *in vivo*. Inhibiting the interaction with any component of the complement system means reducing the probability of MAC formation, characterizing a way to evade the immune system (Meri et al., 2005; Fraga et al., 2011; Daroz et al., 2021).

Considering the production of the FecR proteins in *Lb*P, our findings may suggest that LIC_20185 is more readily recognized by components of the human complement system or other serum components. This could explain the reduced survival of *Lb*P pMG.LIC_20185.6xhis when exposed to active NHS, a finding that does not support a role for LIC_20815 in resistance to complement. Another possible explanation involves differences in the tertiary structures of the proteins resulting from their production in different heterologous systems. The purified rLIC_20185 protein used in the interaction assay and C9 polymerization inhibition assays was produced in *E. coli*, while in the survival experiment LIC_20185 was produced in *Lb*P. These two bacteria may fold the protein differently, potentially altering its tertiary structure and, consequently, its function. Furthermore, post-translational modifications, if present, may also differ between hosts and contribute to functional discrepancies.

Furthermore, the wild-type human C9 present in the NHS may also differ structurally from the recombinant human C9 protein used in the assays, possibly presenting a higher affinity for FecR proteins. Even so, the amount of FecR proteins produced by recombinant *Lb*P under the control of the GroES promoter are unknown and may not be sufficient to reproduce the results observed with purified proteins. When the leptospires were incubated with iNHS, a higher survival index is observed, supporting the hypothesis that complement factors are responsible for the reduction in the survival rate observed.

In the host’s bloodstream, pathogenic leptospires can interact with other important plasma proteins, such as VT, and PLG. VT is a multifunctional glycoprotein which is responsible for negatively regulating the complement system, preventing C9 polymerization and therefore MAC formation (Preissner, 1991; Schvartz et al., 1999). For a pathogen, interacting with this protein would contribute to adhesion and resistance to the action of the complement system (Milis et al., 2008; da Silva et al., 2015). In this context, the affinity of the rLIC_20185 protein for VT was very similar to that observed for the adhesin LipL21 (Takahashi et al., 2021).

Other *L. interrogans* FecR proteins, rLIC_11122 (Figueredo et al., 2017) and rLIC_11834 (Domingos et al., 2012), were also able to interact with PLG in a dose-dependent and saturable manner and this interaction also seems to be mediated by the FecR domain, strengthening the importance of this domain for protein-PLG interaction. The interaction of these proteins with PLG occur through kringle domains and converted into PLA in the presence of uPA. PLA is a broad-spectrum serine protease capable of degrading components of the ECM, as well as the immune and fibrinolytic systems. When bound to a pathogen’s membrane, this protease has the capacity to cleave antibodies and C3b, decreasing opsonophagocytosis, facilitating the diffusion of the bacteria out of the bloodstream (Vieira et al., 2009, 2011). Thus, the *in vitro* data obtained suggest that the evaluated FecR proteins could play a key role in the subversion of this host protease system and, consequently, in the pathogenicity of *L. interrogans*, contributing to the stages of immune system evasion and bacterial dissemination to target organs through the sequestration of the host PLG/PLA system. Other human pathogens, such as *Candida albicans* (Crowe et al., 2003) and bacteria of the genus *Streptococcus* (Jones and Holt, 2007), *Mycobacterium tuberculosis* (Xolalpa et al., 2007), and the spirochetes *Borrelia burgdorferi* (Klempner et al., 1995; Koenigs et al., 2013) and *Treponema denticola* (Tegels et al., 2018) possess proteins capable of interacting to PLG generating PLA, indicating their possible role in the virulence of these agents.

This work reinforces the limitations of using simplified systems and heterologous hosts, such as *Lb*P, as a surrogate organism to study complex host-pathogen interactions. It also highlights the importance of investigating and integrating the activity of recombinant proteins in more complex biological and environmental contexts, to achieve a deeper understanding of leptospirosis pathogenesis.

## Conclusion

Our findings suggest that LIC_10902 and LIC_20185 may participate at the interface between *L. interrogans* and its host. *In vitro* assays revealed that rLIC_20185 binds to host tissue molecules elastin and E-cadherin, while both proteins could contribute to immune evasion by trapping C8, C9 and PLG, probably through the FecR domain, thus preventing the polymerization of the C9 component. Their roles in pathogenesis are reinforced by their aa sequence conservation in virulent strains. However, the fact that production in *Lb*P did not confer protection against human serum highlights the multifactorial nature of leptospiral virulence. These discrepancies suggest that the full functionality of these proteins may depend on a native context, involving a precise membrane topology to synergistic cooperation with other virulence factors that simplified, *in vitro* models cannot fully replicate.

## Data Availability

The original contributions presented in the study are included in the article/**Supplementary Material**, further inquiries can be directed to the corresponding author/s.

## Supplementary material

Supplementary Figure 1 Dose-response assay between rLIC_20185 and host molecules. One µg of host-components were immobilized in 96 well ELISA plates and incubated with different rLIC_20185 concentrations. Detection was carried out using mouse-derived hyperimmune serum (1:5,000), followed by incubation with peroxidase-conjugated anti-mouse IgG antibody (1:5,000). The graphs shown correspond to one representative independent experiment from a duplicate.

Supplementary Figure 2 Dose-response assay between rLIC_10902 and host molecules. One µg of host-components were immobilized in 96 well ELISA plates and incubated with different rLIC_10902 concentrations. Detection was carried out using mouse-derived hyperimmune serum (1:5,000), followed by incubation with peroxidase-conjugated anti-mouse IgG antibody (1:5,000). The graphs shown correspond to one representative independent experiment from a duplicate.

Supplementary Table 1 Compilation of URLs, date of access, software version used. NS: not specified at the website link.
