# Supplementary material for: Host interaction functions of LIC_10902 and LIC_20185 highlight the multifactorial nature of Leptospira interrogans pathogenesis
Source: Front Cell Infect Microbiol. 2026 Oct 16;16:1988027. doi: 10.3389/fcimb.2026.1988027 (PMC13719327; doi:10.3389/fcimb.2026.1988027)
Supplement: Supplementary file 1 [file Supplementaryfile1.pdf]

Supplementary Table 1. Compilation of URLs, date of access, software version used.

Software	Version	Link	Access date
ProtParam	NS	https://web.expasy.org/protparam/	Sep/2026
PFAM (InterProScan)	6	https://www.ebi.ac.uk/interpro/entry/pfam/	Sep/2026
SMART	10	https://smart.embl.de/	Sep/2026
CELLO	NS	http://cello.life.nctu.edu.tw/	Jun/2023
PSORTb	3.0.3	https://psort.org/psortb/	Jun/2023
TMHMM	2.0	https://services.healthtech.dtu.dk/services/TMHMM-2.0/	Sep/2026
LipoP	1.0	https://services.healthtech.dtu.dk/services/LipoP-1.0/	Sep/2026
SignalP	6.0	https://services.healthtech.dtu.dk/services/SignalP-6.0/	Sep/2026
PSIPRED	4.0	https://bioinf.cs.ucl.ac.uk/psipred/	Nov/2025
WebCutter	2.0	https://heimanlab.com/cut2.html	Jul/2023
PyMOL	2.5.4	https://pymol.org/	Jul/2026
AlphaFold	v1.5.3	https://colab.research.google.com/github/sokrypton/ColabFold/blob/main/AlphaFold2.ipynb#scrollTo=kOblAo-xetgx	Dec/2023

NS: not specified at the website link
